# Therapeutic landscape of primary refractory and relapsed diffuse large B-cell lymphoma: Recent advances and emerging therapies

**DOI:** 10.1186/s13045-025-01702-5

**Published:** 2025-07-01

**Authors:** Allison M. Bock, Narendranath Epperla

**Affiliations:** Huntsman Cancer Institute at the University of Utah, Salt Lake City, UT USA

**Keywords:** Diffuse large B-cell lymphoma, Large B-cell lymphoma, Relapsed/refractory lymphoma

## Abstract

Diffuse large B-cell lymphoma (DLBCL) is an aggressive, yet curable malignancy, that has had practice changing treatment approvals in both the frontline and relapsed setting in the last 5 years. Advent of novel therapeutic options in the recent years has added greater complexity in treatment selection and optimal sequencing given multiple treatments with the same therapeutic target or immunotherapeutic mechanism of action. Key features impacting treatment selection include the timing of relapse, eligibility for curative options in the second line setting, including chimeric antigen receptor T-cell therapy (CAR-T) and autologous stem cell transplant (auto-SCT), as well as considerations of mechanism of action and side effect profile. This article provides a comprehensive review on recently approved therapies for relapsed or refractory DLBCL, emerging cellular and non-cellular therapies, and a summary of our approach to the management of these patients.

## Introduction

Diffuse large B-cell lymphoma (DLBCL) is the most common aggressive non-Hodgkin lymphoma (NHL) and can be cured in 60–70% of patients with frontline immunochemotherapy [1–3]. Despite the recent approval of polatuzumab-vedotin in combination with rituximab, cyclophosphamide, doxorubicin, and prednisone (Pola-R-CHP) for high risk patients (International Prognostic Index [IPI**]**, score of 2 or greater) based on a 6.5% two year progression free survival (PFS) benefit compared to R-CHOP, no overall survival (OS) benefit has been shown and there does not appear to be an improvement in the proportion of patients who develop primary refractory disease or early relapse compared to R-CHOP [4]. For the 30–40% of patients who relapse following frontline immunochemotherapy, treatment remains challenging, and outcomes are often poor given the aggressive nature of this disease and the need to choose treatment in the context of the patient’s other medical conditions. Prior to 2017, standard treatment for all patients was therapy with platinum-based chemotherapy regimens and consideration for high dose chemotherapy (HDT)/auto-SCT with few palliative chemotherapy options for transplant-ineligible patients [5–7]. The multicenter retrospective Scholar-1 study has been used as a benchmark of outcomes for patients with relapsed disease, reporting a median OS of only 6.3 months in patients refractory their last line of therapy [8]. Historically, over 80% of patients failed to respond sufficiently to second line chemotherapy or were ineligible to receive auto-SCT, leaving only 20% of patients cured in the relapsed setting with auto-SCT [7–9]. In 2017, CD19-directed chimeric antigen receptor T-cell therapy (CAR-T) with axicabtagene ciloleucel (axi-cel) for DLBCL patients who were either refractory to second line chemotherapy or relapsed after autologous stem cell transplant demonstrated an overall response rate (ORR) of 82% and complete response (CR) rate of 58% with a 5 year OS of 42.6%, confirming the curative potential of this cellular therapy in the third line (3L) or later setting [10, 11]. This approval was followed shortly after by two other CD19-directed CAR products. In 2021, CAR-T with either axi-cel or lisocabtagene maraleucel (liso-cel) were FDA approved for patients with primary refractory disease or disease relapsing within 12 months as an alternative and preferred second line treatment option instead of auto-SCT [12, 13].

**Fig. 1:**
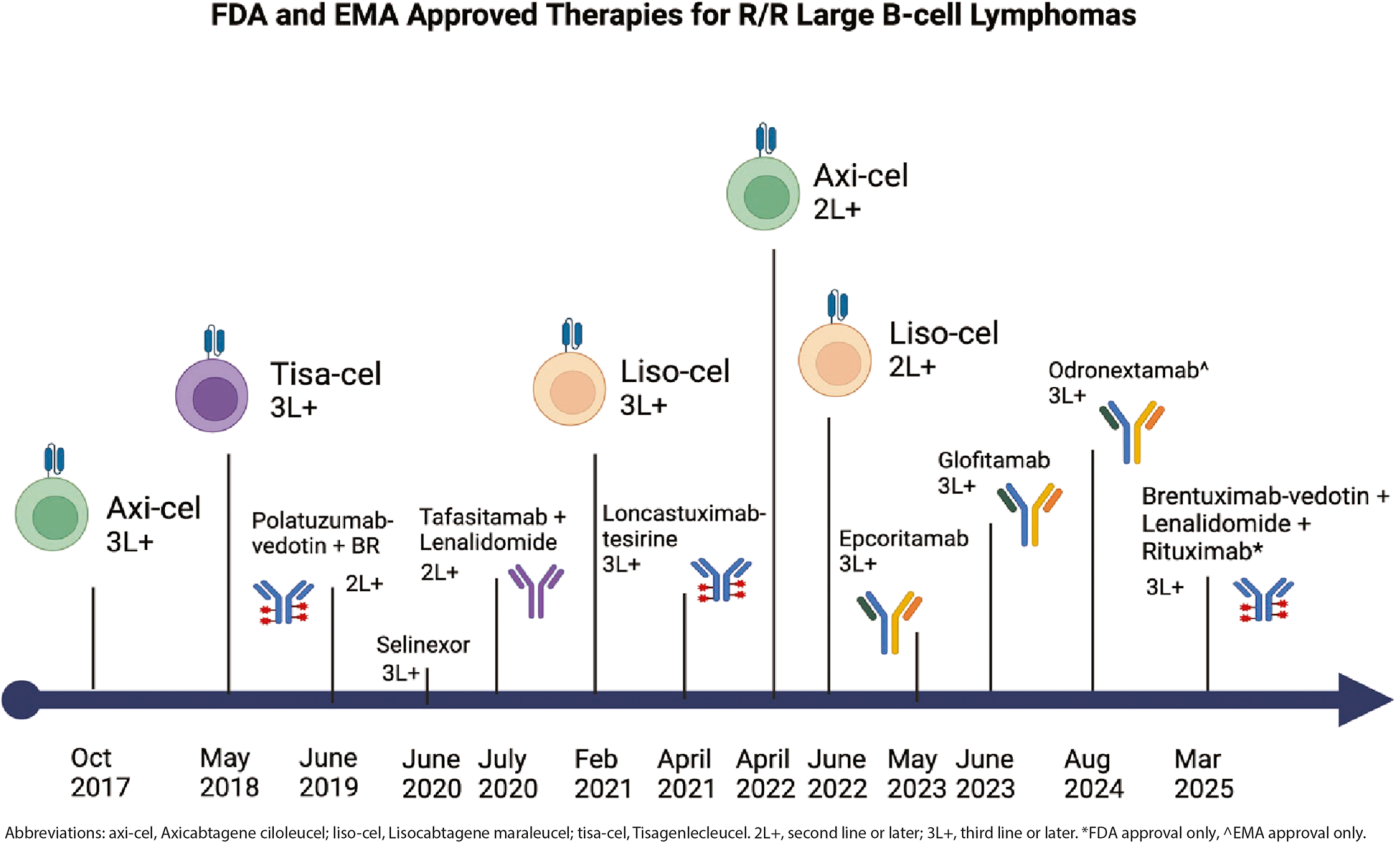
FDA and EMA approved novel treatments and cellular therapies for relapsed/refractory DLBCL

Concurrently, there has been development and approval of multiple targeted therapies and combination treatment options for patients that has broadened the treatment landscape for relapsed/refractory (R/R) DLBCL and has led to remarkable, durable remissions for subsets of relapsed patients that have not been seen outside of the few patients responding to auto-SCT in the past (Fig. 1). While this data is promising, there are still many unanswered questions on optimal treatment selection and sequencing as all of these therapies have been approved in the last 5 years with no head-to-head comparisons, though there is emerging evidence in real world data sets. Additionally, all of the newer approved therapies can lead to a durable CR for a subset of patients, with the majority typically relapsing within 6 months of initiation. We do not yet have reliable biomarkers for treatment selection, with the most powerful prognostic indicator being response to therapy. The goal of this review is to provide a comprehensive overview of recently approved treatments for patients with relapsed or refractory DLBCL. We will also review single agent and combination treatment strategies of both cellular and non-cellular therapies that are currently under development in clinical trials. We will review the current role of cellular therapy and provide an overview of our management of patients with relapsed/refractory DLBCL.

## Approved novel treatments for relapsed DLBCL

**Fig. 2:**
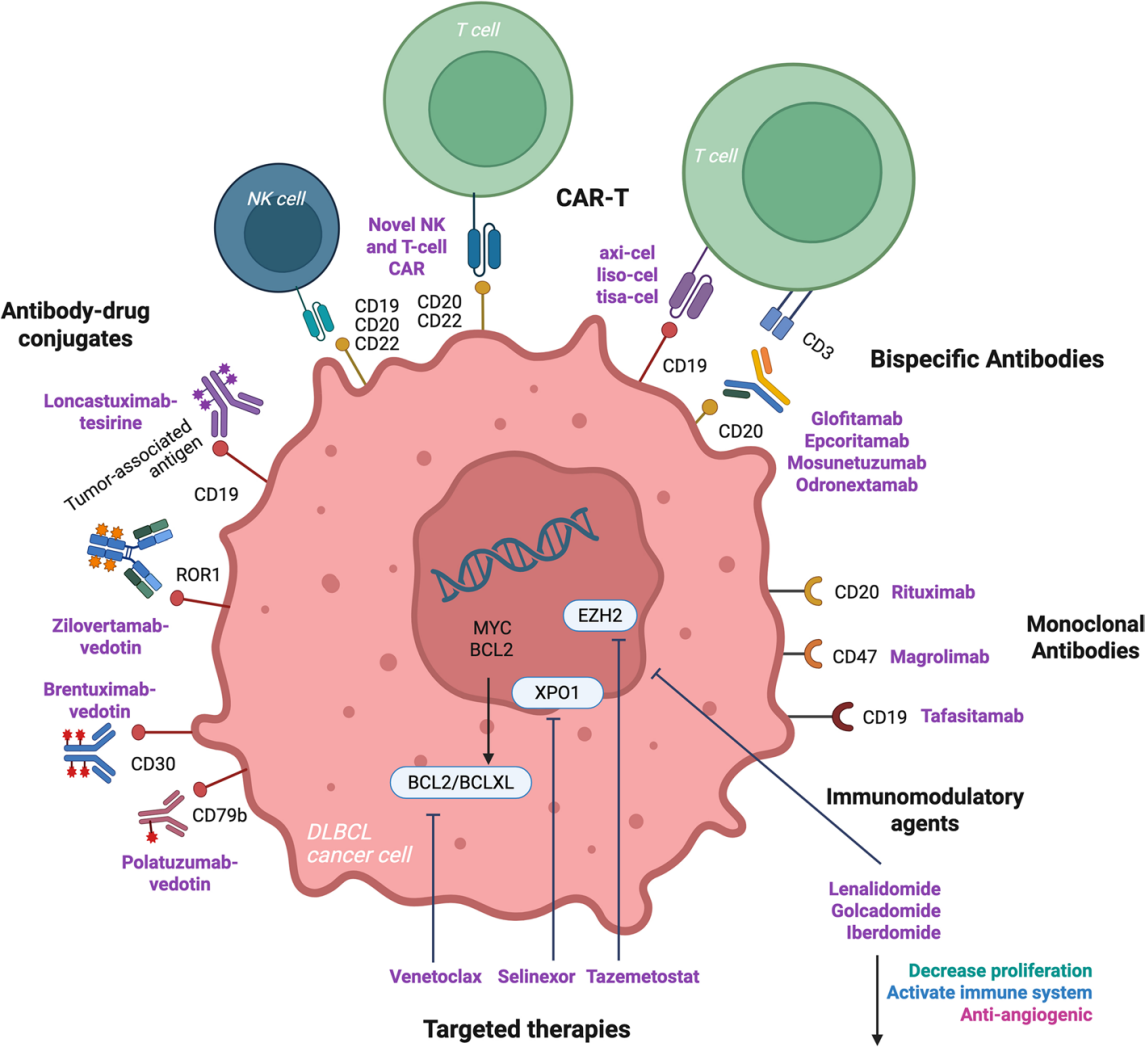
Therapeutic targets of interest in DLBCL

The recent FDA and EMA approved therapies in R/R DLBCL (Fig. 1) are therapies with immunotherapeutic or novel mechanisms of action targeting key B-cell pathways, which are improving outcomes compared to historical cytotoxic chemotherapy (Fig. 2). Odronextamab has received EMA, but not FDA approval, while brentuximab-vedotin in combination with lenalidomide and rituximab (R2) has only FDA approval.

### Bispecific Antibodies

Bispecific antibodies (BsAbs) were designed as an off-the-shelf immunotherapeutic approach to activate the effector T-cell response against malignant lymphoma B-cells. Unlike CAR-T, BsAbs are being developed to target two and sometimes three different antigens. The currently approved BsAbs epcoritamab, glofitamab, and odronextamab target CD20 on malignant B-cells and CD3 on T-cells. The molecular structure of each of these constructs is similar to an immunoglobulin G (IgG) antibody, which is an improvement upon the initial bispecific t-cell engager structure which consisted of two single-chain variable fragments with two binding domains. The IgG like structure of BsAbs have improved the half-life of these therapies and has less neurotoxicity given decreased central nervous system (CNS) penetration.

### Epcoritamab

Epcoritamab is a IgG1 BsAb with a 1:1 ratio of CD20 and CD3 binding that is administered subcutaneously and continuously until disease progression or toxicity. The CD20 epitope binding domain is shared with ofatumumab but unique from both rituximab and obinutuzumab. Epcoritamab is given weekly for three 28-day cycles, followed by every 2 week dosing for 6 cycles, before transitioning to monthly administration. The risk for cytokine release syndrome (CRS) is mitigated with two step-up doses before the first full dose. Dexamethasone pre-treatment and for 3 days after is required for the first three doses, and with subsequent treatment until a full dose is given without CRS or immune effector cell-associated neurological syndrome (ICANS) events. In the EPCORE-NHL-1 study a heavily pre-treated patient population (*n* = 148) with 40% having received prior CAR-T, the ORR/CR rate was 61%/38% (Table 1) [14]. While the median PFS is 4.4 months in the overall population, a subset of patients achieving a CR have sustained durable remissions. In patients achieving a CR, the median duration of complete response (DoCR) is 36.1 months with a median PFS of 37.3 months and the median OS has still not been reached at a median follow up of 31 months [15]. CRS of any grade was observed in 51% (*n* = 80) with a grade (G) 3 event in 2.5% (*n* = 4) and no grade 4 or 5 events. ICANS occurred in 6% of patients (5.7% G1/G2, 0.6% G5). Other notable adverse events (AEs) include injection site reactions (G1/G2 19.7%), neutropenia (any grade 21.7%, Grade ≥ 3 14.6%), and anemia (any grade 17.8%, Grade ≥ 3 10.2%). CRS and ICANS adverse events have been further mitigated with additional supportive care measures in cycle 1 including pre and post intravenous hydration in additional to 3 days of dexamethasone [16].

**Table 1:** Efficacy of recently approved novel treatments for patients with R/R DLBCL

Treatment	MOA	N	LOT	Prior LOT	Pts with primary refractory disease (%)	Prior CAR-T, N (%)	Treatment schedule (dosing/duration)	ORR/CR rate (%)	Median PFS (months)	Median DOR (months)	Median duration of CR (months)	PFS estimates	OS estimates	Notable treatment emergent adverse events	Reference
Tafasitamab + Lenalidomide	CD19 MAb + CELmod	81	2L +	2	19	0	Lenalidomide: 20 mg orally on Day 1–21 for 12 cycles; Tafasitamab: IV 12 mg/kg QW C1-C3; Q2 W C4 and onward	57.5/40	11.6	43.9	Not reached	12 month PFS of 50%	24 month OS of 90.6%^a^	neutropenia, peripheral edema, diarrhea, cough	[17, 18]
Pola-BR	ADC + chemotherapy	192	2L +	2	64	1 (1)	IV on Day 1 and 2, every 21 days for 6 cycles	56.6/52.8	6.6	9.5	NR	24 month PFS of 28.4%^b^	median OS 12.5 months	Neuropathy, infections, neutropenia, thrombocytopenia, anemia	[19, 20]
Loncastuximab-tesirine	CD19 ADC	145	3L +	3	20	14 (9.7)	IV 150ug/kg every 21 days for C1-C2, 75ug/kg C3 onward	48.3/24.8	4.9	13.4	13.4	12 month PFS of 82.9%^c^	median OS 9.5 months	neutropenia, elevated GGT, peripheral edema, photosensitive rash	[21, 22]
Epcoritamab	CD20 x CD3 BsAb	148	3L +	3	61	61 (38)	step up dosing C1D1 and C1D8 prior to target dose of 48 mg C1D15; QW C1-3; Q2 W C4-C9; Q4 W C10 onward	59/41	4.4	20.8	36.1	24 month PFS of 65.4%^d^	24 month OS of 76.2%^d^	CRS, ICANS, neutropenia, infections	[14, 15]
Glofitamab	CD20 x CD3 BsAb	155	3L +	3	58	51 (33)	QW step up dosing C1; 30 mg C2-C12 Q3 W	52/40	4.9	18.4	29.8	24 month PFS of 62.9%^d^	24 month OS of 74.6%^d^	CRS, ICANS, neutropenia, tumor flare, infections	[23, 24]
Odronextamab	CD20xCD3 BsAb	127	3L +	3	55	NR^e^	Step up dosing C1; QW 160 mg C2-C4; Q2 W 320 mg until POD	52/31.5	4.4	10.5	36.3	Median PFS of 20.4^a^	12 month OS of 48%	CRS, ICANS, infections	[25]
BV-R2 vs R2	ADC + CELmod	230	3L +	3	57	32 (29)	BV: 1.2 mg/kg IV Q3 W; Lenalidomide: 20 mg PO daily; R 37 mg/m2 IV Q3 W	64/42	4.2	8.3	18.9	Median PFS 4.2 months	Median OS 13.8 months	Peripheral neuropathy, diarrhea, neutropenia, thrombocytopenia	[26]
Selinexor	XPO1 inhibitor	127	3L +	2	NR	NR	60 mg orally twice weekly	28/12	2.6	9.3	NR	NR	median OS 9.1 months	thrombocytopenia, neutropenia, emesis, hyponatremia	[27]

*Abbreviations: MOA* mechanism of action, *N* number of patients, *LOT* line of therapy, *Mab* monoclonal antibody, *Pts* patients, *Pola* polatuzumab vedotin, *BR* bendamustine and rituximab, *R2* rituximab and lenalidomide, *R* rituximab, *CELmod* cereeblon E3 ligases, *ADC* antibody–drug conjugate, *BsAb* bispecific antibody, *2L* second-line, *3L* third-line, *LOT* line of therapy, *CAR-T* chimeric antigen receptor T-cell therapy, *NR* not reported, *C* cycle, *D* day, *QW* every week, *Q2 W* every 2 weeks, *Q3 W* every 3 weeks, *ORR* overall response rate, *CR* complete response, *PFS* progression free survival, *DOR* duration of response, *OS* overall survival, *POD* progression of disease, *CRS* cytokine release syndrome, *ICANS* immune effector cell-associated neurological syndrome

^a^among patients in a CR

^b^from primary analysis, rest of efficacy data from most recent analysis

^c^among patients in CR (36)

^d^among pts in CR

^e^outcomes for patients post CAR-T are reported in ELM-1 study

### Glofitamab

Glofitamab is an IgG1 BsAb with a 2:1 configuration of CD20 and CD3 and a flexible linker that enhances target-effector binding. The CD20 binding epitope is identical to obinutuzumab; the bivalent binding to CD20 allows for administration of other anti-CD20 agents and this BsAb was developed with Obinutuzumab pre-treatment 1 week before the first step up dose to mitigate CRS. Glofitamab was developed with two step up doses prior to reaching the first full dose on cycle 2 (C2) day 1 (D1). Glofitamab is given intravenously (IV) once every 3 weeks for a fixed duration of 12 cycles. In a similarly heavily pre-treated population (*n* = 155), the ORR/CR rate was 52%/40% with a median PFS of 4.9 months [23](Table 3). With longer follow up, the median DoCR is 29.8 months with 57.3% of patients in a CR at 24 months among patients achieving a CR at end of treatment [24]. Longer follow up has not been reported for the overall population. CRS of any grade was seen in 63% (*n* = 97) with grade ≥ 3 in 4% of patients (*n* = 6). ICANS occurred in 8% of patients (*n* = 12; 5% G1/G2, 3% Grade ≥ 3) [23]. Other notable AEs include neutropenia (38%), anemia (31%), infection (38%), and tumor flare (3%). Infectious prophylaxis for herpes virus reactivation is important and most academic centers are also administering pneumocystis jirovecii pneumonia (PJP) prophylaxis and monitoring IgG levels with intravenous immunoglobulin (IVIG) replacements, similar to post CAR-T protocols for all patients on BsAb.

Real-world studies of epcoritamab and glofitamab used in the standard of care setting are limited but starting to emerge. The multicenter retrospective study REALBITE included 209 patients treated with epcoritamab or glofitamab, and included a higher percentage of double-hit and triplet lymphoma (DHL/THL, DLBCL with *MYC* and *BCL2* rearrangements) (20.2%) and prior CAR-T (60.3%) then were included in the registrational studies [28]. The response rates were lower than reported in the clinical trials with an ORR/CR rate of 50.6%/23.8%. Response rates were also significantly inferior in patients with absent CD20 expression on pre-treatment biopsy highlighting CD20 as an important biomarker of response [28]. Grade ≥ 3 CRS/ICANS events were slightly higher than have been reported [28], and other real world studies have identified bone marrow involvement, low albumin and low absolute neutrophil count (ANC) as risk factors associated with increased risk of CRS [29]. Lower response rates have also been demonstrated with earlier administration after CAR-T [30]. Post CAR-T administration of both epcoritamab and glofitamab did not occur prior to day 100 in the clinical trials. Thus, these real-world studies reflect the administration in a broader and more high-risk patient population that would likely not have been trial eligible due to laboratory criteria, medical comorbidities and earlier relapse post CAR-T. They also emphasize the importance of assessing CD20 status prior to administration based on currently available data. Overall, both epcoritamab and glofitamab are promising therapies that have demonstrated similar, high response rates in challenging patient populations. However, these real-world studies show that patients with DHL and early relapse post CAR-T likely still have lower response rates and remain a challenging patient population to treat. Additional data on clinical features as well as biomarkers of response and resistance are needed to select patients most likely to benefit from this therapy. There is also growing interest in moving bispecific antibodies to earlier lines of therapy and combining with other active agents to improve upon clinical activity.

### Odronextamab

Odronextamab is a fully human IgG4-based CD20xCD3 bispecific antibody [25]. Promising phase 1 data were initially presented by Bannerji et al. for R/R NHL [25], leading to a pivotal phase 2 study currently enrolling for 5 different disease groups (NCT03888105, NCT02290951). For aggressive B-cell NHL, odronextamab is given in a step-up regimen in C1 (0.7 mg, 4 mg, 20 mg), followed by a weekly dose of 160 mg for C2-C4, followed by 320 mg every 2 weeks until disease progression or unacceptable toxicity. Patients with a CR ≥ 9 months are transitioned to monthly dosing. In the primary analysis of the phase 2 ELM-2 trial which excluded patients with prior CAR-T (*n* = 127), the ORR and CR rate were 52.0% and 31.5%, respectively, at a median follow up of 29.9 months [31]. The median DOR was 10.2 months and median duration of CR was 17.9 months. The most common TEAEs were CRS (53.3%), anemia (38.6%), and neutropenia (30.7%), similar to other CD20xCD3 bispecific antibodies. The majority of CRS events were low grade (51.7% G1/G2, 1.7% G3) with the 3 dose step up regimen. No ICANS events were reported with the current step-up regimen. Patients with DLBCL and prior CAR-T therapy (ELM-1 cohort) (*n* = 44) had an ORR of 48% and a CR rate of 30% [25].

## Antibody–drug conjugates (ADCs)

ADCs are emerging as another promising treatment for R/R DLBCL, with three currently approved therapies – Polatuzumab-vedotin, loncastuximab-tesirine, and brentuximab-vedotin. ADCs allow the targeted delivery of a potent cytotoxic molecule intracellularly, thereby limiting systemic toxicity.

### Polatuzumab combinations

Polatuzumab vedotin (Pola) targets CD79b which is a common cell surface receptor on most B-cells, including in DLBCL [32]. Pola is conjugated to monomethyl auristatin E (MMAE) which blocks mitosis through the inhibition of tubulin polymerization [33]. This potent mitotic inhibitor is delivered directly into cells after endocytosis of the polatuzumab/CD79b complex [33]. Pola received its first approval in transplant-ineligible patients with R/R DLBCL in combination with bendamustine and rituximab (Pola-BR) [19, 20]. Pola is given IV at 1.8 mg/kg in combination with BR on day 1, followed by bendamustine on day 2, for a total of six 21-day cycles. In the initial phase 1/2 study, pola-BR demonstrated an ORR/CR rate of 70%/57.5%, a median PFS of 6.3 months and median OS of 12.4 months [19](Table 1). Similar outcomes have been observed in real world studies of Pola-BR or Pola-R [34–36]. The most notable adverse events are neuropathy (43.6%) [19], which is mainly grade 1–2 and infrequently leads to discontinuation. Grade ≥ 3 infections (23.1%), neutropenia (46.2%), anemia (28.2%) and thrombocytopenia (41%) are also observed [19], with cytopenias being mainly from bendamustine rather than pola based on comparative studies that included treatment with Pola-R and Pola-BR [35, 36]. Mutations of CD79b are observed in 23% of activated b-cell (ABC) subtype R/R DLBCL [37], yet, the initial studies of Pola-BR showed clinical efficacy regardless of cell of origin [20, 38]. A recent retrospective multicenter study reported that ORR and CR rate were higher among patients with ABC subtype R/R DLBCL compared to germinal center b-cell (GCB) subtype R/R DLBCL [39].

### Loncastuximab combinations

Like many other targeted therapies in the relapsed setting, loncastuximab-tesirine (lonca) targets the common B-cell surface marker CD19. Lonca is a humanized anti-CD19 IgG1 antibody that is conjugated to pyrrolobenzodiazepine, that once internalized into the cell, causes DNA damage and inhibits cell growth. Lonca is administered IV, starting at a dose of 150ug/kg every 21 days for the first 2 cycles followed by 75 ug/kg every 21 days from cycle 3 onward until progression of disease or toxicity. In the first phase 1 study of lonca in patients with R/R B-cell NHL (*n* = 88), the ORR/CR rate was 59.4%/40.6% and most common treatment emergent adverse events included neutropenia, thrombocytopenia, edema, rash and elevated gamma-glutamyl transferase (GGT). The phase 2 clinical trial LOTIS-2 in R/R DLBCL (*n* = 145) demonstrated an ORR of 48.6% and CR rate of 24.1% [22]. The median duration of response (DOR) was 12.6 months, median PFS was 4.9 months, and median OS was 9.9 months [22]. Updated results with longer follow up are similar (Table 1). Durable responses were achieved in patients with high-risk disease features such as DHL/THL. Responses were observed at all levels of CD19 expression by IHC [40]. Patients with bulky disease (≥ 10 cm) were excluded from the phase 2 based on lower response rates in the initial phase 1 study. Treatment emergent adverse events (**TEAEs**) were noted in nearly all patients (99%) and similar to the phase 1 trial, the most common of these were cytopenias (neutropenia 40%, neutropenic fever 3%, thrombocytopenia 33%, and anemia 26%), elevated GGT (40%), and peripheral edema (20%) with the most common grade ≥ 3 adverse events being neutropenia, anemia and elevated GGT [22]. Treatment was discontinued in 23% of patients due to TEAEs, 51% had dose delays and 8% needed dose reductions [21, 22].

In the largest real-world analysis (*n* = 187) that included high proportions of primary refractory disease (32%) and prior CAR-T (60%), single agent lonca demonstrated an ORR and CR rate of 33 and 14%, respectively [41]. Median PFS was 2.1 months and median OS was 4.6 months. Similar to the initial phase 1 study, no patients with bulky disease (> 10 cm) had an objective response. Response rates before and after CAR-T were similar [41]. Lonca has also been evaluated in combination with rituximab for patients with DLBCL, as reviewed in bridging therapy section.

### Brentuximab combinations

Brentuximab-vedotin (BV) is a CD30-directed monoclonal antibody that is conjugated to the microtubule-disrupting agent MMAE by a protease-cleavable linker and leads to anti-tumor activity by inducing cell cycle arrest and apoptosis [42]. BV mediated cytotoxicity is also enhanced by immune cell stimulation and bystander effects. As a single agent, BV had an ORR of 44% in R/R DLBCL with variable CD30 expression [43]. Generally, CD30 expression greater than 1% is considered positive and associated with better response to therapy. Notably, responses can be achieved in patients without CD30 expression likely due to the potency of the drug at lower levels of expression that are below the threshold of detection for immunohistochemistry (IHC) [44]. BV was studied in combination with lenalidomide and rituximab (R2) in the phase 3 Eschelon-3 study (*N* = 230), for patients with R/R DLBCL, which included 29% with prior CAR-T [26]. At a median follow up of 15.5 months, the combination of BV with R2 demonstrated an ORR of 64%, a CR rate of 40% with a median PFS of 4.2 months and median OS of 13.8 months [26]. The R2 arm had an ORR of 42%, CR rate of 19%, and median PFS and OS of 2.6 months and 8.5 months, respectively. Treatment with BV + R2 demonstrated statistically significant differences in all efficacy outcomes compared to R2 alone. Interestingly, response rates were similar in the CD30 + population (defined as > 1%) and CD30- population. Most common TEAE in the BV + R2 vs R2 arm included neutropenia (46% vs 32%), anemia (29% vs 27%), and diarrhea (31% vs 23%). Peripheral neuropathy was higher in the BV + R2 arm (any grade 20%, G3 4%) [26].

## Small molecule inhibitors

### Selinexor

Selinexor is an oral selective inhibitor of XPO1-mediated nuclear export, which leads to reductions in *MYC* and *BCL2* oncogenes. This therapy was FDA approved after 2 prior lines of therapy based on the phase 2b SADAL trial in R/R DLBCL patients with 2–5 prior lines of therapy (*n* = 127) [27]. Patients received Selinexor 60 mg on days 1 and 3 weekly until disease progression or unacceptable toxicity. The ORR was 28% with a 12% CR rate and median PFS of 2.6 months [27] (Table 1). The most common TEAEs included thrombocytopenia (61%), nausea (58%), fatigue (47%), anemia (43%), and diarrhea (35%). TEAEs led to dose modification in 70% and discontinuation in 17%. While not quantified well in terms of AEs, this therapy is difficult to tolerate and given the change in the therapeutic landscape since this approval in June of 2020, we do not recommend this therapy for most patients.

## Non-cellular immune therapies

### Tafasitamab-Lenalidomide

Tafasitamab (Tafa) is an immunotherapeutic drug with a CD19-targeting Fc-enhanced humanized monoclonal antibody that mediates antibody-dependent cellular cytotoxicity, direct cytotoxicity, and antibody-dependent phagocytosis [17]. Lenalidomide (Len) is an immunomodulatory agent that exhibits anti-tumor activity by modulation of cereblon E3 ubiquitin ligase, inducing cell cycle arrest, inhibiting secretion of pro-inflammatory cytokines, stimulating proliferation of T-cells and natural killer (NK) cells, and inhibiting angiogenesis [45]. The combination of Tafa and Len was initially studied in the pivotal phase 2 study L-MIND for patients with 1–3 prior lines of therapy and ineligible for auto-SCT [17]. Patients receive tafasitamab IV weekly in 28-day cycles for C1-C3, then every 2 weeks during cycles 4–12. Lenalidomide is administered on days 1–21 of cycles 1–12. After cycle 12, patients in CR continued to receive tafasitamab every 2 weeks until disease progression or toxicity. After 35 months of follow up in 80 patients, the ORR was 57.5% and CR rate was 40% [18] (Table 1). The median DOR was 43.9 months with a median PFS of 11.6 months and median OS of 33.5 months. Among patients achieving a CR, the median OS has not been reached and the OS at 24 months was 90.6%. The most common TEAE (all grades) included neutropenia (51%), anemia (37%), thrombocytopenia (31%), diarrhea (36%), cough (27%), and peripheral edema (24%). The most common grade ≥ 3 adverse events included neutropenia (49%), thrombocytopenia (17%) and febrile neutropenia (12%). A temporary interruption of tafasitamab or lenalidomide occurred in 79% and 35% of patients, respectively, with majority of cases due to AEs. Dose reductions of lenalidomide occurred in 46% of patients.

Initially, patients with primary refractory disease defined as lack of CR or relapse within 6 months were excluded from enrollment. Even after inclusion later with an amendment, this patient subgroup still only represented 18% of patients (*N* = 15). Not surprisingly, real world studies of Tafa-Len have showed lower ORR/CR rates and PFS when including all relapsed patients [46–48].

## Autologous stem cell transplant (auto-SCT)

Determining eligibility for either auto-SCT or CAR-T is a key first step for patients with R/R DLBCL as both of these represent curative treatment options. Medical comorbidities appear to impact patient outcomes following auto-SCT more than chronologic age [49], however many transplant centers still have an age cut off of 70 or 75. Auto-SCT may still be considered for select fit patients > 70. Eligibility criteria for auto-SCT also more strictly requires adequate cardiac, pulmonary, renal, and hepatic function given the need to tolerate high dose chemotherapy. The hematopoietic cell transplantation-specific comorbidity index (HCT-CI) score was developed to assess risk post allogeneic transplantation but can also predict non relapse mortality (NRM) after auto-SCT [50]. This risk score includes the impact of other medical conditions such as autoimmune disease, obesity, and diabetes on complication rate and NRM.

For patients relapsing greater than 12 months following completion of first-line therapy, the current standard of care for eligible patients remains platinum-based chemotherapy [7, 51] followed by auto-SCT should they achieve chemosensitivity which can lead to a cure for approximately 20% of all relapsing patients, and has been the standard of care treatment for all R/R DLBCL patients for the past 20 years [5–7, 52, 53]. Among patients proceeding to auto-SCT, approximately 40–50% will experience a durable remission [7, 48]. Long term outcomes following auto-SCT are consistently better for patients achieving a pre-transplant CR compared to a partial response (PR) [7, 54, 55]. For patients achieving a PR (Deauville score of 4 or 5), the 3 year PFS was 49% compared to 77% for patients achieving a CR (Deauville score 1–3) [54]. If patients are ineligible for auto-SCT based on institutional guidelines, or if they fail to achieve a CR with second line (**2L**) platinum-based chemotherapy, CAR-T would a reasonable option regardless of timing of relapse [53].

Auto-SCT may be considered in select patients with early relapse should they achieve chemosensitivity (CR or PR) based on the data from CIBMTR [56–58]. Primary refractory patients generally have chemoresistant disease with poor responses to platinum-based therapy limiting the role of auto-SCT which hinges on chemosensitivity for clinical benefit [38, 59]. While some patients with a PR may still have good outcomes with 2L auto-SCT, this appears to be best for the younger patient (< 65) [60], without primary refractory disease, and with low tumor burden on pre-auto-SCT PET-CT.

Allogeneic stem cell transplant is another curative treatment option for eligible patients in certain circumstances such as those progressing after auto-SCT or CAR-T or after auto-SCT in areas where CAR-T is not available [53]. Discussion on allogeneic transplant is beyond the scope of this article.

## Chimeric antigen receptor T-cell therapy (CAR-T)

CAR T-cell therapy is an adoptive cell therapy with genetically modified T-cells that express a chimeric antigen receptor, an engineered receptor with an extracellular tumor antigen (such as CD19) binding domain, a transmembrane domain, and an intracellular T-cell signaling domain. CAR-T cells induce T-cell activation leading to destruction of malignant B-cells. Anti-CD19 CAR-T products (axi-cel, liso-cel, and tisagenlecleucel [tisa-cel]) were initially FDA approved for R/R DLBCL in the third line setting [11, 61, 62] and more recently (axi-cel and liso-cel) in the second line setting [12, 63]. For the purpose of this review, DLBCL also encompasses primary mediastinal LBCL (PMBCL), transformed follicular lymphoma (FL)/marginal zone lymphoma (MZL), and FL grade 3B, which were all included in the following studies. All three are engineered with a CD3Z activator domain, but axi-cel has a CD28 costimulatory domain which is unique from liso-cel and tisa-cel which have a 4-1BB costimulatory domain, which were developed to increase CAR-T cell persistence. Each of these therapies follow a similar process of leukapheresis, CAR-T genetic engineering and manufacturing, and 3 days of lymphodepleting chemotherapy with fludarabine and cytarabine prior to infusion of CAR T cells. The most notable adverse events with CAR-T include cytokine release syndrome, neurotoxicity, risk for infections, B-cell aplasia and hypogammaglobulinemia. In this section, we will discuss eligibility for CAR-T, bridging therapy, use in 2L versus 3L and beyond, post-CAR-T relapse mitigation strategies and emerging CAR-T therapies.

### Eligibility

Eligibility for CAR-T is different from auto-SCT and is increasing the cure rate for healthy older patients in particular with multiple real-world studies confirming similar safety and efficacy in patients > 70 years [64–67]. Assessments of ability to complete activities of daily living (ADLs) and independent ADLs (IADLs) and Cumulative Illness Rating Scale-Geriatric (CIRS-G) scoring in addition to physical functioning and formal geriatric assessment are important considerations in the assessment of older adults with lymphoma for CAR-T [68, 69]. In terms of organ function, real-world studies have also confirmed that similar safety and efficacy can be achieved in patients who did not meet the strict trial eligibility of the pivotal trials [70–72]. This was corroborated in the PILOT study that evaluated liso-cel for patients who were considered transplant ineligible, but CAR-T eligible, regardless of timing of relapse [73].

There have also been multiple case studies addressing the safety and efficacy of CAR-T in human immunodeficiency virus (HIV) patients [74–77]. While the data is limited, there was similar efficacy and did not appear to be an increased risk of infections and similar rates of CRS/ICANS. It is recommended but not required to have a CD4 count > 200 and undetectable HIV viral load. However, CAR-T has been safely conducted in patients with a CD4 count ranging from 52–629 and detectable HIV viral load [74–76, 78]. These studies have all reported successful CAR-T manufacturing with axi-cel, alleviating suspicions that low CD4 counts may lead to manufacturing failure.

### Bridging Therapy

Many patients require bridging therapy for disease control during the CAR-T manufacturing process. This typically refers to therapy given after apheresis but can also include treatment given while waiting for apheresis. No standard bridging therapy has been established, as the duration of disease control depends on both patient and disease features as well as system-specific factors. The time for referral to a cellular therapy expert, completion of pre-CAR testing, insurance approval and leukapheresis can vary widely from center to center, even for patients starting in an academic medical system. The risk of disease progression while waiting for CAR-T remains a key barrier for treatment. In the initial study of tisa-cel, 92% of patients required bridging therapy, with 30% of enrolled patients discontinuing prior to CAR T-cell infusion due to disease progression [62]. Bridging therapy has not been shown to negatively impact ORR, PFS or OS in most studies, but rather reflects the higher proportion of high-risk patients needing both bridging and CAR-T [79, 80]. Additionally, growing evidence supports that lower tumor burden, and even absence of detectable disease, prior to CAR-T is associated with better outcomes and less CRS and ICANS adverse events [81–85]. Current bridging treatment strategies utilized include steroids, immunochemotherapies (chemo backbone includes gemcitabine, etoposide, and platinum agents [cisplatin/oxaliplatin]), antibody–drug conjugates (polatuzumab-vedotin-based regimens and loncastuximab-tesirine), radiation, and targeted agents (Tafa-Len). Bridging therapy with chemotherapy can lead to prolonged cytopenias following CAR-T [80] and often has limited durable efficacy given prior exposure to chemotherapeutic agents and chemoresistance, especially in primary refractory patients [38, 47]. Bendamustine is associated with persistent cytopenias and use of this therapy prior to apheresis has been associated with difficult T-cell collection as well as lower ORR, PFS and OS post CAR-T [86]. A washout > 9 months has been associated with better outcomes post CAR-T [86]. We recommend avoiding bendamustine if possible, prior to CAR-T. Key considerations for choice of bridging therapy include timing of relapse, prior frontline therapy, need for rapid response, mechanism of action, and impact on T-cell apheresis and manufacturing.

**A. Radiation therapy.**

Radiation therapy (RT) has become a key bridging strategy prior to CAR-T given its efficacy in chemo-resistant patients and association with better post CAR-T outcomes in some studies [79, 87–90]. RT has been shown to facilitate presentation of tumor-associated antigens for the priming of T cells, stimulating the adaptive immune system and enabling anti-tumor immune responses [91]. RT has demonstrated high ORRs (67–100%) as bridging therapy, which is similar to what has been observed for R/R DLBCL in prior studies [79, 92]. When compared to systemic therapy alone, RT has demonstrated an improved ORR, CR rate, and PFS [79]. Different radiation strategies have been reported including focal RT to a dominant bulky mass or comprehensive to all sites of disease, and shorter courses of low dose RT (2 Gy × 2 Gy) [73, 82, 93].

**B. Antibody–drug conjugates**.

The antibody–drug conjugate Pola in combination with rituximab has been utilized as a successful, chemotherapy-free, bridging strategy to CAR-T with ORR rates of 42–54% and CR rates of 21–40% and is another leading treatment approach supported by real-world studies [19, 34, 47]. As mentioned, for bridging therapy, Pola-R with omission of bendamustine is recommended. An additional consideration for use of Pola-based regimens at relapse is the recent approval of Pola in combination with R-CHP as standard of care frontline treatment for DLBCL. The efficacy in relapsed patients with prior exposure is likely to be less – especially those with primary refractory disease or early relapse.

Lonca with or without rituximab has demonstrated ORR and CR rates of 40–63% and 13–50%, with better ORR and CR rates seen in combination with rituximab and as second line/bridging therapy compared to later lines of therapy with a dedicated bridging study of lonca-R ongoing (NCT06788964) [47, 94, 95].

**C. Small molecule inhibitors**.

Tafa-Len has demonstrated an ORR of 26–40% and CR rate of 11–15% in real-world studies of patients treated in 2L + setting [46, 47, 96]. Tafa-Len use prior to CAR-T has been avoided given concern that Tafa treatment may reduce efficacy to subsequent CD19 CAR-T through antigen loss or epitope masking as there is partial overlap between 4G7 (Tafa CD19 target) and FMC63 (approved CAR-T CD19 target). Preclinical data have shown that prior tafasitamab does not affect subsequent CAR-T therapy in mouse models, and showed that conversely, prior tafasitamab may enhance CAR-T therapeutic index and reduce CRS through the transient occupancy of CD19 epitope masking [97]. Several retrospective studies have also demonstrated detectable CD19 expression following tafasitamab discontinuation [98, 99] and successful administration of CAR-T [100].

**D. Bispecific antibodies.**

Bispecific antibodies are being explored as a bridging therapy but, similar to the concern with anti-CD19 agents, there is concern these therapies may negatively impact the T-cell repertoire prior to CAR administration. If used as bridging, it is also generally recommended to start after apheresis. A study by the DESCAR-T registry demonstrated no difference in safety or efficacy parameters in patients who received BsAb as bridging prior to CAR-T (including patients who receive both pre and post apheresis) compared to cohort of patients who were BsAb naïve [101]. As we anticipate approvals for both epcoritamab and glofitamab in the 2L setting in combination with chemotherapy, further studies will be needed to assess sequencing before CAR-T.

**E. CD19-targeted agents prior to CD19 CAR-T**.

Use of CD19-directed agents prior to CAR-T (Tafa/Len or lonca) has generally been avoided out of concern that they may reduce CD19 antigen expression and reduce CD19 CAR-T efficacy. There is still uncertainty on whether this is the best approach, but a few small retrospective studies have reported successful administration of CAR-T and subsequent CR after CD19-targeted therapies (tafa-len, lonca, and other investigational therapies) [95, 96, 100, 102, 103]. These studies have not found a significant decrease in CD19 expression after CD19-targeted therapy, though the numbers remain too small to draw definitive conclusions.

### Second Line Therapy CAR-T

Three separate clinical trials have evaluated CD19 CAR-T compared to standard of care (SOC) with platinum-based chemotherapy followed by auto-SCT for patients with primary refractory or early relapsing DLBCL defined as relapse less than 12 months from completion of frontline chemoimmunotherapy. In an international, phase 3 randomized study, ZUMA-7 (*n* = 359), axi-cel has demonstrated both an event free survival (EFS) and OS benefit for CAR-T in the second line setting compared to auto-SCT. At a median follow up of 24.9 months, the median EFS was 8.3 months in the axi-cel arm and 2.0 months in the SOC arm (auto-SCT), with a 2 year EFS rate of 41% and 16%, respectively (Table 2) [12]. With an additional 47.2 months of follow up, the estimated 4 year OS was 54.6% and 46.0% for axi-cel compared to auto-SCT (hazard ratio (HR) 0.73, *p* = 0.03), despite a high rate of off protocol cellular therapy administration (57%) in the SOC arm [63]. Most notable grade 3 or greater adverse events included CRS (6%), ICANS (21%), neutropenia (69%), anemia (30%), and thrombocytopenia (15%) (Table 2) [12].

**Table 2:** Safety and efficacy of approved CAR therapies for R/R DLBCL

**Treatment**	**N**	**Phase**	**Construct**	**CRS, any grade, %**	**CRS grade >3, %**	**ICANS, any grade, %**	**ICANS grade >3, %**	**TRAE Grade >3, %**	**ORR, %**	**CR, %**	**median DOR (months)**	**PFS estimate**	**Median PFS (months)**	**Median OS (months)**	**Reference**
**Second Line Therapy**															
Axi-cel	359	3	CD19, 4-1BB costimulatory domain	92	6	60	21	91	83	66	NR	2 year PFS of 46%	14.7	Not reached	[12, 63]
Liso-cel	184	3	CD19, CD28 costimulatory domain	49	1	12	4	92	86	66	Not reached	36 month 50.9%	10.1	Not reached	[61, 104, 106]
**Third Line**															
Axi-cel	111	2	same as above	93	13	64	28	95	83	59	11.1	15 month PFS of 41%	5.8	Not reached	[10, 11]
Liso-cel	340	1/2	same as above	42	2	30	10	79	73	53	Not reached	12 month PFS of 44.1%	6.8	21.1	[61, 106]
Tisa-cel	93	2	same as above	64	22	21	12	89	52	40	Not reached	12 month PFS of 40%	NR	8.3	[62]

*Abbreviations: N* number, *CRS* cytokine release syndrome, *ICANS* immune effector cell-associated neurological syndrome, *TRAE* treatment-related adverse events, *ORR* overall response rate, *CR* complete response rate, *PFS* progression free survival, *OS* overall survival, *NR* not reported, *axi-cel* Axicabtagene ciloleucel, *liso-cel* Lisocabtagene maraleucel, *tisa-cel* Tisagenlecleucel

In the pivotal TRANSFORM randomized phase 3 trial evaluating liso-cel compared to SOC with platinum-based chemotherapy and auto-SCT (*n* = 184), liso-cel demonstrated a significant improvement in EFS with a median EFS of 10.1 months in the liso-cel arm compared to 2.3 months in the SOC arm (HR 0.35, *p* = 0.0001) with a median follow of 6.2 months(Table 2.) [13]. With longer follow up of 33.9 months, the estimated 3 year OS rate was 62.8% for liso-cel compared to 51.8% for auto-SCT (HR 0.757), with 66% of patients in the SOC arm crossing over to receive liso-cel [104]. Any grade of CRS and grade 3 CRS were observed in 49% and 1% (no grade 4 or 5 events), respectively. Any grade of ICANS and grade 3 ICANS were observed in 12 and 4% (no grade 4 or 5 events), respectively (Table 2). Based on these results, both liso-cel and axi-cel were FDA approved in 2022.

The international, multicenter phase 3 randomized study BELINDA (*n* = 322) was the third study of second line CAR-T for R/R DLBCL that evaluated tisa-cel compared to SOC with platinum-based chemotherapy and auto-SCT. At a median follow up of 10 months, the median EFS in both groups was 3 months (HR 1.07, *p* = 0.61) [105]. Safety was comparable to TRANSFORM and Zuma-7. Based on the lack of efficacy, tisa-cel is not approved in the 2L.

### Third Line and Beyond

The three CD19 directed CAR-T products that are currently approved for relapsed/refractory DLBCL after 3 or more lines include axi-cel, liso-cel and tisa-cel. In 2017, CAR-T with axi-cel (Zuma-1) for patients who were either refractory to platinum-based chemotherapy or relapsed after autologous stem cell transplant demonstrated an ORR of 82% and CR rate of 58% (Table 2) [10, 11].

Long term follow up of Zuma-1 (axi-cel), which has the longest follow up to date, has demonstrated a 5 year OS rate of 42.6%, disease specific survival rate of 51.0%, and a median duration of CR of 62.2 months [10]. This long term follow up data confirms the curative potential of CAR-T [10].

Liso-cel showed efficacy and manageable safety profile as a third line or later treatment for patients with R/R DLBCL in the TRANSCEND NHL 001 trial [61]. Liso-cel demonstrated an ORR of 73% and a CR rate of 53% [61]. Longer follow up has reported a 5 year OS of 38.1%, disease specific survival rate of 52.0%, and a median duration of CR of 26.1 months [106]. Any grade CRS occurred in 42% (*n* = 113) of patients with grade 3 or higher in 2% (*n* = 6). Any grade ICANS occurred in 30% (*n* = 80) of patients with grade 3 or higher in 10% (*n* = 27).

Tisa-cel was studied in a similar patient population as axi-cel and liso-cel in the phase 2 JULIET study (*n* = 93). The ORR was 52% with a 40% CR rate [62]. The estimated 24-month PFS rate was 33%. Frequency and severity of CRS and ICANS for the three products are shown in Table 2..

Several real-world studies have confirmed safety and efficacy of CAR-T therapy in the 2L and 3L setting, many of which included patients who would not have met the inclusion criteria for the pivotal trials based on comorbidities or organ dysfunction [65, 70, 71, 107–110]. The 12-month PFS and OS following CAR-T across these studies were 34–47% and 52–64% [65, 70, 108, 110].

## Post CAR-T relapse mitigation strategies

Many post CAR-T maintenance strategies are currently in clinical development. These strategies have been developed based on the clear inferior outcomes for patients who fail to achieve an early CR to CAR-T compared to those who do achieve CR [111, 112]. In the case of trials targeted at patients with PR, the rationale is to intervene prior to overt relapse given the high risk of relapse in the first 6 months post CAR-T for all patients undergoing CAR-T. The investigational agents being used have a different mechanism of action and the hope is for synergism between any residual CD19 CAR cells and the investigational agent. Many of the approved therapies in the R/R setting are being investigated as post CAR-T relapse mitigation strategies (NCT05633615, NCT06238648, NCT05464719, NCT06552572, NCT06854159), in addition to emerging therapies, some of which are being studied prior to CAR-T (NCT 05934838, NCT05757219).

### Relapse post CAR-T

While initial responses to CAR-T are high and a subset of patients may achieve a cure, 50–60% of patients will not respond or will eventually relapse [11–13, 61, 62]. Several real-world studies have reported dismal outcomes for relapsed patients with a median OS of approximately 5–6 months [113–115]. There are many factors that contribute to relapse that are beyond the scope of this article and have been well described in other reviews [116]. It is important to obtain a biopsy at relapse to confirm disease relapse and assess for CD19 and CD20 positivity. Loss of CD19 expression by antigen escape can be observed in up to 30% of patients [114, 117, 118]. However, the clinically relevant threshold of CD19 expression required for response has not been defined, and may not be detectable with current methods, such as IHC. Consideration for clinical trials is highly recommended. Alternative CAR-T products with dual targeting mechanisms or allogeneic CAR-T may still be effective patients relapsing after commercial CD19 CAR-T. Bispecific antibodies (glofitamab and epcoritamab) both included 30–40% of patients with relapse post CAR-T in their initial phase 1/2 studies. Both demonstrated an ORR of approximately 50% and a CR rate of approximately 35%, similar to the overall patient population. Brooks et al. reported outcomes in the largest non-trial cohort (*n* = 209) with 60% having prior CAR-T therapy and showed lower ORRs and CR rates of 49–53% and 23–25%, respectively, with a median PFS of 2.5 months in patients relapsed post CAR-T [28]. Even though response rates are likely to be lower than reported in the initial clinical trials, bispecific antibodies are a preferred treatment choice post CAR-T given limited immunosuppression compared to other available therapies. However, they do still come with a higher risk of infection and close monitoring is needed. Lonca is another preferred therapy post CAR-T for patients who remain CD19 positive, with an ORR of 46.2% in the LOTIS-2 study [22], an ORR/CR rate of 73%/34% for patients receiving 2L CAR-T/3L lonca and ORR/CR rate of 78%/17% for patients receiving 3L CAR-T/4 th line (4L) lonca in a retrospective study [119]. Several studies have demonstrated worse prognosis for patients with earlier relapse post CAR-T (< 90 days) then later relapse (> 90 days). This appears to impact response rates to subsequent therapies as well [28]. For those achieving a response after CAR-T failure, consideration for auto-SCT or allogeneic stem cell transplant remains a consideration. The 3-year PFS is approximately 30–40% for patients able to achieve a CR prior to allogeneic transplant, though data after CAR-T failure is still limited [120, 121].

### Emerging CAR-T therapies

Several CAR products are under investigation including autologous CAR-T constructs with dual targets or engineering to enhance efficacy or safety, allogeneic CAR constructs that are “off the shelf”, and CAR constructs incorporated into non T-cell immune system cells, most notably NK cells. Newer autologous CAR therapies with dual targeting mechanisms include CD19/CD20 bispecific CAR-T therapies (NCT05098613, NCT05826535, and NCT04792489), CD19/CD22 CAR-T therapies (NCT05972720, NCT03287817) and CD20/CD79 CAR-T therapies (NCT05169489)(Table 3). A newer CD19-directed autologous CAR-T products, rapcabtagene autoleucel, is generated to produce T-cells with a rapid manufacturing process (2 days) and preserves T-cell stemness (NCT03960840) [122]. Many other studies of next generation autologous CAR therapies are also ongoing with editing aimed at enhancing efficacy and reducing toxicity (NCT04684563, NCT06279611).

**Table 3:** Investigative novel CAR treatments for R/R DLBCL

Treatment	NCT#	Target	Phase	Sample size	Median prior LOT	Median follow up (months)	ORR% (CRR%)	Median PFS (months)	PFS estimate	OS estimate	CRS Any grade, % (Grade ≥ 3, %)	ICANS Any grade, % (Grade ≥ 3, %)	Neutropenia Any grade, % (Grade > 3, %)	Thrombocytopenia any grade, % (Grade ≥ 3, %)	Other notable treatment emergent adverse events, %
Zamto-cel	04792489	Auto CD20/CD19	1	27	2	NR	50 (38)	NR	NR	NR	14 (5)	15 (4)	NR	NR	NR
Rap-cabtagene autoleucel	03960840	Auto CD19	2	63	2	16.4	88 (65)	11.9	12 month PFS of 48%	NR	43 (6)	6 (3)	62% G3	25% G3	Infections (G3, 27%)
firicabtagene autoleucel	04088890; 05972720	Auto CD22	1, 2	38	4	36.7	68 (53)	3	3 year PFS of 30%	3 year OS of 47%	93 (0)	10 (0)	100 (100)	86 (52)	second primary malignancies (MDS/AML, 11%), infection, hypogammaglobulinemia
Tak-007	03056339	Allo NK CAR19/IL15	1,2	37	4	12	89 (83)	NR	NR	NR	2.7 (0)	0 (0)	100 (34)	89 (27)	asthenia (35%), dysrhythmia (19%), rash (11%)
CB-010	04637763	Allo CAR19/PD-1 knockout	1	16	2	NR	94 (69)	NR	NR	NR	44 (0)	25 (13)	NR (G3 56)	NR (69)	CRS, ICANS, thrombocytopenia, neutropenia, anemia. No GVHD
FT596	04245722	iPSC-derived NK CAR19	1	20	4	NR	52 (64)	NR	NR	NR	10 (0)	0 (0)	85 (NR)	40 (NR)	nausea (75%), peripheral edema (35%). No GVHD
LY007	06279611	Auto CD20	1	9	3	NR	89 (78)	NR	88.9% at 6 months	100% at 6 months	66 (0)	0 (0)	77% G3	0 (0)	cytopenias
Cemacabtagene ansegedleucel (501/501 A)	03939026 (ALLO-501); 04416984 (ALLO-501 A)	Allo CAR19	1,2	33	4	7.1	67 (58)	NR	NR	NR	24 (0)	0 (0)	NR	NR	prolonged G3 or greater cytopenia (12%), infection, infusion reaction. No GVHD
AUTO3	03287817	Auto CD19/CD22, PD1	1	24	3	NR	57 (29–50)	NR	NR	NR	27 (0)	9 (9)	73 (73)	82 (64)	nausea (36%), headache (36%), cough (27%), infection (G3 18%)
CTX112	05643742	Allo CAR19	1	9	2	NR	67 (44)	NR	NR	NR	44 (0)	22 (0)	NR	NR	No GVHD
huCART19-IL18	04684563	Auto CD19	1	21	7	15	79 (52)	8.7	NR	median OS not reached	71 (14)	14 (0)	NR	NR	fatigue (38), hypotension (29) and low fibrinogen (23)
AB1010	04673617	Allo NK	1,2	17	4	NR	41 (18)	NR	NR	NR	12 (0)	0 (0)	NR	NR	No ICANS or GVHD

*Abbreviations: NK* natural killer cell, *Allo* allogeneic, *Auto* autologous, *iPSC* induced pluripotent stem cell, *LOT* line of therapy, *CAR* chimeric antigen receptor therapy, *NR* not reported, *ORR* overall response rate, *CR* complete response, *PFS* progression free survival, *DOR* duration of response, *OS* overall survival, *CRS* cytokine release syndrome, *ICANS* immune effector cell-associated neurological syndrome, *GVHD* graft-versus-host disease, *G* grade

Allogeneic (allo) CAR therapies have the advantage of being readily available, potentially more cost-effective, and effective in patients who may have impaired T-cells from prior chemotherapy or underlying conditions. A universal CAR-T targeting CD19 (UCART19-ALLO-501) has finished enrolling with promising efficacy for pts with R/R DLBCL (NCT03939026). The allo CAR CTX112 has 5 edits using CRISPR/Cas9 editing to enhance potency and CAR-T persistence(NCT05643742) [123]. Many other studies with allogeneic constructs are underway (Table 3).

CAR-NK cells are being engineered with a similar approach as CAR-T with the benefit that NK cells as part of the innate immune system do not require antigen presentation for anti-tumor efficacy. Clinical trials of CAR-NK targeting CD19, CD20, and CD22 for the broad treatment of B NHLs are underway (NCT03056339, NCT03056339 (TAK-007), NCT05020678, NCT05487651, NCT06707259). Preliminary safety and efficacy for these novel CAR strategies with available data are shown in Table 3.

### Investigational therapies in relapsed setting

Non cellular therapy agents that have not been approved include one other CD20xCD3 bispecific antibody, mosunetuzumab (approved for R/R FL), as well as several other bispecific antibodies with different targets, novel ADCs, immunomodulatory and epigenetic therapies. Details on these therapies are shown in Table 4, however this is not an exhaustive list of all therapies currently under investigation.

**Table 4:** Investigative single-agent treatments for R/R DLBCL

Treatment	NCT#	Phase	Sample size	Prior LOT	Median follow up (months)	ORR, % (CR, %)	Median PFS (months)	PFS estimate	OS estimate	CRS Any grade, % (Grade ≥ 3, %)	ICANS Any grade, % (Grade ≥ 3, %)	Neutropenia any grade, % (Grade ≥ 3, %)	Thrombo-cytopenia any grade, % (Grade ≥ 3, %	Notable Treatment emergent adverse events, (%)	Reference
*Bispecific antibodies*															
Mosunetuzumab	02500407	1/2	88	3	10.1	42 (23.9)	3.2	12 month PFS of 22.6%	12 month OS of 48%	26.1 (2.3)	2.2 (0)	27 (21.6)	3.4 (3.4)	hypophosphatemia (11.4%), infections (12.5% grade 3 or higher), tumor flare (1%)	[124, 125]
AZD0486	04594642	1	51	4	5	43 (33)	NR	NR	12 month OS 77%	34 (0)	11 (5.7)	35 (19)	NR	Hypogammoglobulinemia (18%), hypokalemia (15%)	[126]
Plamotamab	02924402	1	36	4	NR	52.9 (23.5)	NR	NR	NR	72.2 (0)	3.2 (0)	18.2 (16.7)	18.2 (11.1)	hyponatremia (31.8%), hypophosphatemia (25%)	[127, 128]
*Non cellular, immune-based or antibody-based therapies*															
Zilovertamab vedotin	05141841	2	98	3	4.5	29 (13)	2.5	6 month PFS of 15%	6 month OS of 62%	-	-	48 (39)	NR	Peripheral neuropathy (18%), infusion reaction (1%)	[129]
Magrolimab (± Rituximab)	02953509	1/2	99	3	7.9	24 (12)	1.8	12 month PFS of 15%	12 month OS of 44.3%	-	-	20 (15)^a^	18 (12)^a^	infusion reactions (39)	[130]
Golcadomide (± rituximab)	03930953	1/2	77	4	10.2	48 (30)	NR	NR	NR	-	-	62 (59)	18 (7.7)	pneumonia (9%), pruritus (10%)	[131]
Iberomide	04464798	1/2	46	4	NR	55 (32)	NR	NR	NR	-	-	57 (49)	22 (13)	Muscle spasms (11%), asthenia (13%)	[132]

*Abbreviations: NCT* national clinical trial, *ORR* overall response rate, *CR* complete response rate, *PFS* progression free survival, *OS* overall survival, *CRS* cytokine release syndrome, *ICANS* immune effector cell-associated neurological syndrome, *NR* not reported, *pts* patients, *G* grade

^a^estimates only from published data

- not applicable

## Bispecific antibodies

### Mosunetuzumab

Mosunetuzumab is a fully humanized IgG1 CD20xCD3 BsAb developed using knob-in-hole engineering to allow the near similarity to human IgG1 [124], and has been developed in both an IV and subcutaneous formulation. To reduce CRS with initial IV dosing, step-up IV dosing is given weekly for three weeks in the first cycle. Mosunetuzumab in a subcutaneous formulation demonstrated promising single-agent activity in a phase 1/1b in R/R B-cell NHL with an ORR of 44.4% (16/36) and a CR rate of 22.2% (8/36) [124, 133]. These response rates are similar to an earlier study with IV dosing (ORR 37.1%; CR 19.4%) [124]. A single arm phase 2 expansion cohort (*n* = 88) of IV mosunetuzumab demonstrated similar ORR/CR rates [125]. The 12 month PFS and OS estimates were 22.6% and 48% in this study [125] (Table 4). Durable responses were achieved with a median duration of response of 20.4 months. Notable TEAEs include CRS, ICANS, neutropenia, and hypophosphatemia, with similar incidence to epcoritamab/glofitamab. Despite promising activity, mosunetuzumab has not been approved as a single agent for R/R DLBCL, though several combination trials are ongoing with other agents.

### Plamotamab

Plamotamab is another CD20 x CD3 bispecific antibody that is being evaluated with IV and subcutaneous administration (NCT02924402) [127, 128]. Similar to other BsAbs, plamotamab was administered in a step-up dosing regimen to reduce the risk of CRS and neurotoxicity, with all observed events being limited to grade 1 and 2 (Table 4). Among a heavily pre-treated patient population, the ORR and CR rate were 47.4% and 26.3%, respectively. Results from the phase 1 dose escalation study for part D with subcutaneous administration included 15 patients with R/R DLBCL. In the DLBCL cohort that included 82% of patients with prior CAR-T, the ORR and CR rate were 52.9% and 23.5%, respectively, with dose escalation ongoing.

### AZD0486

AZD0486 is a CD19 x CD3 BsAb uniquely designed to bind CD3 with low affinity to reduce cytokine release upon T-cell activation, while preserving effective T-cell cytotoxicity against malignant B cells. Updated phase 1 dose escalation results from the study in heavily pre-treated patients with DLBCL have been reported (NCT04594642) [126]. The median prior number of lines of therapy was 4, 59% had prior CAR-T, and 24% had bulky disease. A total of 51 patients have been randomized, with 40% being efficacy evaluable (received doses ≥ 2.4 mg). In this cohort, the ORR and CR rate were 43% and 33%, respectively. Higher response rates were observed in patients receiving the target dose of 7.2 mg (*n* = 19) with ORR/CR of 47%/42% and who were CAR-T naïve (*n* = 5) with an ORR and CR rate of 80%.

Grade 3 and 4 AEs observed in ≥ 10% of pts with DLBCL who received the two step up dose regimen were neutropenia (26%) and anemia (17%). No treatment-related deaths or AEs leading to discontinuation occurred. Among patients who received a two step-up dose regimen (*n* = 35), CRS events occurred in 12 (34%) patients (all grade 1), grade 1–2 ICANS occurred in 4 (11%) patients, and grade 3 ICANS occurred in 2 (5.7%) patients (0/2 at target dose). All CRS and ICANS events were fully reversible and without sequelae, limited to cycle 1, and did not lead to treatment discontinuation [126].

### FS118

FS118 is a novel tetravalent IgG1 bispecific antibody that simultaneously targets two immune checkpoints, PD-L1 and LAG-3. In a Phase 1 study, previously conducted in both US (IND Number:137281) and Europe (EudraCT Number: 2021–002946-33), FS118, was well tolerated up to dosage 20 mg/kg IV QW with no dose limiting toxicities (DLT) observed. This established the recommended Phase 2 dose (RP2D) of 10 mg/kg IV QW and this dose was investigated in FS118-21,201 study (EudraCT 2021–003406-47) enrolling non-small cell lung cancer patients (Cohort A, *n* = 21) and DLBCL patients (Cohort C, *n* = 10). In the phase 2 open-label basket study in a cohort of DLBCL patients (*n* = 10), the ORR/CR rate were both 20% [134]. No checkpoint inhibitor has been approved for R/R DLBCL, despite initial enthusiasm. Whether targeting with a bispecific antibody, rather than a single PD-L1 agent, will be more effective remains to be seen.

## Antibody–drug conjugates

Zilovertamab vedotin is a ROR1-targeting ADC that has been evaluated in the phase 2 waveLINE-004 study (NCT05141841) for patients with R/R DLBCL (*n* = 98) [129]. Most patients received > 3 lines of therapy (71%) and were ineligible for auto-SCT or CAR-T (58%). Among 98 patients, the ORR was 29% and the CR rate was 13%. The median PFS was 2.5 months, with a median OS of 10.6 months. The most common TEAEs were neutropenia (48%), anemia (26%) and peripheral neuropathy (18%).

## Non-cellular immune therapies

### CELMod Therapies

Newer novel cereblon E3 ligases (CELMoD) therapies such as iberdomide and golcadomide are also under investigation (Table 4).

Golcadomide is a novel CELMoD that has more efficient generation of the closed conformation of the cereblon complex than lenalidomide, leading to deeper and more rapid degradation of Ikaros/Aiolos, which are crucial for B-cell malignancy development. Patients enrolled in this phase 1/2 study received different dose schedules of golcadomide with and without rituximab (NCT03930953) [131]. Treatment is being investigated using two different dose levels (0.2 mg, *n* = 39; 0.4 mg, *n* = 38 in combination with rituximab. Most frequent TEAEs were hematologic (Table 4). The median DOR was 8.3 months, including a durable response > 12 months for 8 patients (10%). Similar response rates were observed in both ABC and GCB cell of origin DLBCL subtypes.

Iberdomide (CC-220) is another newer oral CELMoD agent. In vitro studies suggest iberdomide enhances T-cell immunostimulatory and direct cytotoxic effects compared to lenalidomide. A phase 1/2 study as monotherapy or in combination with rituximab in patients with R/R NHL, including 18 patients with R/R DLBCL, is ongoing (*n* = 46)(NCT04464798) [132]. Among response-evaluable patients (*n* = 38), the ORR and CR rate were 55% and 32%. Most common TEAE of any grade and grade 3 and greater were primarily hematologic (Table 4).

### CD47 targeting

CD47 is an immune checkpoint that is expressed in normal cells and upregulated in various NHLs (DLBCL, mantle cell lymphoma, and FL) [135, 136]. CD47/SIRPα mainly regulates innate immune cell activity and sends out a “do not eat me” signal to escape the attack of innate immune cells [137]. Pre-clinical studies have shown that *MYC* can upregulate the expression of CD47 and that CD47 may place a role in extranodal lymphoma progression [138, 139]. The anti-CD47 antibody, magrolimab (Hu5 F9-G4), combined with rituximab (MR), showed promising activity in a phase 1b study in patients with R/R NHLs (NCT02953509). Among 22 patients (*n* = 15 with DLBCL) the ORR was 50% and CR rate was 36% and the RP2D of 30 mg/kg was identified. Three year follow up in the R/R DLBCL subgroup (*n* = 132) has recently been reported. At a median follow up of 7.9 months, the ORR and CR rate were 24% and 12%, respectively, in the MR subgroup (*n* = 99) [130]. Higher response rates were seen in MR + gemcitabine and oxaliplatin (GemOx) subgroup (*n* = 33). The median PFS was 1.8 months, with a DOR of 9.3 months and median OS of 9.2 months. The median time to response was 1.8 months. Better outcomes in the MR + GemOx arm are likely in part due to the slow time to response with MR alone. The most common AEs were anemia and infusion-related reactions, with most adverse events being grade 1 or 2 [140].

## Combination therapies in relapsed setting

### Combinations of individually active agents

There are numerous ongoing studies evaluating combinations of immunotherapeutics and immunomodulating agents such as bispecific antibodies and Tafasitamab-lenalidomide, cytotoxic chemotherapy, and approved ADC therapies Polatuzumab vedotin and Loncastuximab tesirine. Preliminary data for these combinations is very encouraging. Key considerations for the future will involve assessing synergism and whether these promising agents are best given as single sequential treatments or combination therapies. Additionally, close monitoring for toxicity with combinations will be important, especially for use in older patient populations which is the subgroup that has the most potential to benefit from non-cellular therapy approaches if the clinical activity is promising. Table 5 lists safety and efficacy for the combination treatment strategies discussed in this review. This is not an exhaustive list of all combination treatment strategies, however.

**Table 5:** Investigative combination treatment strategies in R/R DLBCL

Treatment	NCT#	Phase	Sample size	Median prior LOT	Median follow up (months)	ORR, % (CR, %)	Median PFS (months)	PFS estimate	OS estimate	CRS Any grade, % (Grade ≥ 3, %)	ICANS Any grade, % (Grade ≥ 3, %)	Neutropenia any grade, % (Grade ≥ 3, %)	Thrombocytopenia any grade, % (Grade ≥ 3, %	Notable Treatment emergent adverse events, (%)	Ref-erence
Epcoritamab + R-DHAX	04663347	1/2	29	2	27.5	100 (86)	NR	24 month PFS of 60%	24 month OS of 86%	45 (0)	3.4 (0)	48 (NR)	76 (NR)	No new safety signals	[141]
Epcoritamab + GemOx	04663347	1/2	103	2	13.2	85 (61)	11.2	12 month PFS of 44%	12 month OS of 59%^a^	51 (1)	2 (1)	65 (57)	73 (59)	infections (72%)	[142]
Mosunetuzumab + polatuzumab vs R-Polatuzumab	03671018	2	79	2	23.9	78 (58)	11.4	12 month PFS of 64.2%	12 month OS of 73.8%	10 (0)	2.5 (0)	40 (30)	25 (4^)b^	injection site reactions (55%), peripheral neuropathy (27.5%), tumor flare (7.5%)	[143]
Glofitamab + Polatuzumab	03533283	1b/2	129	2	28.2	78.3 (59.7)	12.3	24 month PFS of 41.8	24 month OS of 54.3	44 (1.6)	3.8 (0.8)	42 (33)	15 (10)^b^	peripheral neuropathy (24%), tumor flare (7%)	[144]
Glofitamab + R-ICE	05364424	1b	41	1	NR	83.3 (66.7)	NR	NR	NR	49 (0)	0 (0)	35.7 (28.6)	52.4 (26.8)	Infections (G3, 5%), TLS (G3, 5%)	[145]
Glofitamab + GemOx vs GemOx	04408638	3	274	1	20.7	68.3 (58.5)	13.8	12 month PFS of 51.7%c	24 month OS of 52.8	44 (2)	2 (1)	42 (NR)	48 (NR)	Nausea (49%), peripheral neuropathy (36%)	[146]
Loncastuximab + Venetoclax	05053659	1/2	16	4	NR	57 (43)	NR	NR	NR	-	-	45 (25)	10 (0)	ALT/AST increased (12%), atrial fibrillation 12.5%), elevated bilirubin (18%)	[147]
Zanubrutinib + Lenalidomide	04436107	1	66	2	16.5	58 (42)	5.5	12 month PFS of 34%	median OS not reached	-	-	57 (NR)	15 (NR)	hypokalemia (11%)	[148]
Gloftamab + Englumafusp alpha	04077723	1/2	134	3	16.2	68.6 (56.6)	9.7	1 year PFS of 82.6	median OS of 20.4 months	55.2 (0.7)	0.7 (0)	41 (30)^b^	30.6 (20)^b^	Infections (64%), hepatotoxicity (21%)	[149]
VIPOR	03223610	1b/2	60	3	40	54/38	NR	2 year PFS of 34%	2 year OS of 36%	-	-	70 (52)	90 (45)	diarrhea (68%), hypokalemia (66%), rash (35%), hypophosphatemia (32%), hypomagnesemia (32%), atrial fibrillation (5%)	[150]

*Abbreviations: NCT* national clinical trial, *ORR* overall response rate, *CRR* complete response rate, *PFS* progression free survival, *OS* overall survival, *CRS* cytokine release syndrome, *ICANS* immune effector cell-associated neurological syndrome, *NR* not reported, *pts* patients, *G* grade, *TLS* tumor lysis syndrome, *R-DHAX* rituximab, dexamethasone, cytarabine, oxaliplatin, *GemOx* gemcitabine and oxaliplatin, *R-ICE* rituximab, ifosphamide, carboplatin, etoposide, *BV* brentuximab vedotin, *R2* rituximab and lenalidomide, *VIPOR* venetoclax, ibrutinib, prednisone, obinutuzumab, and lenalidomide

^a^OS for US population

^b^estimates only based on available data

^c^by IRC assessment

- not applicable

### Bispecific antibodies and chemotherapy

While there was initial skepticism about the combination of bispecific antibodies with cytotoxic chemotherapy, preclinical work has demonstrated that BsAb can still be activated with low numbers T-cells thereby allowing combination with T-cell cytotoxic chemotherapy agents and other anti-CD20 antibodies [151, 152]. Glofitamab has been combined with GemOx as second line chemotherapy in the second or later setting for transplant ineligible patients (StarGlo). Patients received 8 cycles of glofitamab-GemOx plus 4 additional cycles of glofitamab monotherapy or R-GemOx alone for a total of 8 cycles [146]. With a median follow up of 20.7 months, this combination demonstrated an ORR and CR rate of 68.3%/58.5%, median PFS of 13.8 months and significant overall survival benefit with a median OS of 25.5 months compared to 12.9 months in patients who received GemOx alone (HR 0.62, *p* = 0.006)(Table 5) [146]. While encouraging data, it is worth noting that most of the patients were enrolled outside of the US where access to CAR-T is not universally approved as standard of care in second line. Very few patients had bulky disease (12.6%) or prior CAR-T therapy (7.1%), which may explain part of the benefit when compared to the single agent activity. Ongoing studies in combination with other common chemotherapy regimens are also underway, including glofitamab in combination with rituximab, ifosphamide, carboplatin and etoposide (R-ICE) (NCT05364424). This phase 1b study in patients intended to proceed to CAR-T or auto-SCT pts (*n* = 42) studied glofitamab combined with 3 cycles of R-ICE. In a population that included 31% of primary refractory patients, the ORR and CR rate were 83.3% and 66.7%, respectively [145]. Similar response rates were seen in the primary refractory subgroup, which is promising. The most common TEAEs of any grade were CRS (57%), thrombocytopenia (53%), anemia (48%), and nausea (36%). CRS and ICANS events were all grade 1 and 2. Only 4 patients had AEs leading to treatment discontinuation.

Epcoritamab has also been combined with the platinum-based regimens R-DHAX and GemOx for transplant eligible patients. In the initial phase 1/2 study, epcoritamab-R-DHAX has demonstrated a ORR and CR rate of 100% and 86% [141]. epcoritamab in combination with GemOx was evaluated as a single arm in a transplant eligible population similar to StarGlo, but notably included higher risk and more heavily pre-treated patients including those with HGBCLs (10%), transformed DLBCL (23%), relapse after auto-SCT (10%), prior CAR-T (28%), primary refractory disease (52%) and bulky disease (19%). At a median follow up of 13.2 months, epcoritamab-GemOx had an ORR and CR rate of 85% and 61% by independent review committee (IRC) assessment [142]. Median PFS was 11.2 months, and median OS was 21.6 months. TEAEs were similar to the initial phase 2 study with manageable rates of CRS and ICANS. The most common TEAEs were thrombocytopenia (59%), neutropenia (57%), and anemia (43%). Phase 3 registrational studies are ongoing (Table 5).

### Bispecific antibodies and ADCs

Several studies are ongoing to assess the synergistic potential of combining a CD20xCD3 bispecific antibody with an ADC. These combinations of approved single agents are highly anticipated and have the potential widen the therapeutic landscape for patients who are transplant or CAR-T ineligible, as well as relapsed post CAR-T.

Both mosunetuzumab and glofitamab have been combined with Pola in patients with R/R DLBCL (Table 5). In the initial phase 1 studies, the ORR/CR rate for mosunetuzumab-pola and glofitamab-pola were 59%/46% [153] and 78%/56% [144], respectively. No new safety signals were seen. In an updated analysis of glofitamab-pola in patients with R/R DLBCL (DLBCL, NOS, PMBCL, DHL/THL, transformed indolent lymphoma) (*n* = 129), the ORR/CR rate were maintained at 78.3% and 59.7% respectively. The median DOR was 26.4 months and median PFS was 12.3 months. The study included 34% of patients with DHL/THL (*n* = 29) and encouragingly these patients had similar response rates compared to the overall population with a CR rate of 65.9%. Very few patients (10%) were treated with subsequent therapies.

Similarly, subcutaneous mosunetuzumab in combination with Pola has demonstrated similar response rates and outcomes. This combination was further investigated in a phase 2 study compared with Pola-R for patients with ≥ 1 prior LOT (NCT03671018)(n = 79). The ORR and CR rate were 77.5% and 57.5% in the mosunetuzumab-Pola arm compared to 50% and 35% in the Pola-R arm [143]. The median PFS was not reached and 6.4 months for mosunetuzumab-Pola vs Pola-R. No new safety signals were seen. CRS and ICANS events were grade 1 or 2, with no grade ≥ 3 events observed. This combination is being further explored in the phase 3 SUNMO study for transplant-ineligible DLBCL (NCT05171647). The key question for these ongoing trials is if the clinical efficacy will be the same in patients treated with Pola-R-CHP as frontline therapy. In both of these studies, a few patients had prior Pola-R-CHP, but no data has been published on response rates in the subgroup of patients.

Both mosunetuzumab and glofitamab are also being investigated in combination with loncastuximab-tesirine (LOTIS-7) for transplant ineligible patients with R/R DLBCL in second line or greater setting (NCT04970901) and there is an ongoing study of mosunetuzumab and loncastuximab for patients with R/R DLBCL after ≥ 2 lines of therapy (NCT05672251). These studies combining a CD19-directed ADC with a CD20xCD3 bispecific antibody have the potential to be highly effective given unique mechanisms of action.

### Combinations of active therapies with investigational agents

Epcoritamab, mosunetuzumab and glofitamab are currently in clinical trials with novel agents for R/R DLBCL including trials with monoclonal antibodies and PDL1 inhibitors (mosu + tiragolumab ± atezolizumab), Bruton tyrosine kinase inhibitors (BTKi) (glofitamab + poseltinib/lenalidomide, epcoritamab + lenalidomide ± ibrutinib), and in combination with other novel bispecific antibodies (glofitamab + englumafusp alfa, glofitamab + RO7443904) and novel CELMoDs (mosu/glofitamab + iberdomide or golcadomide).

### Bispecific antibody combinations

Glofitamab is being investigated in combination with englumafusp alpha in patients with R/R DLBCL(n = 134)(NCT4077723) [149]. Englumafusp alfa is an antibody-like fusion protein that simultaneously targets CD19 on B cells and 4-1BB on T cells and other immune cells. Englumafusp alpha boosts T cell effector functions and prevents T-cell anergy.

This chemo-free combination hopes to augment T-cell activity and anti-tumor efficacy of glofitamab. TEAE were seen in 90.3% of patients. CRS of any grade was observed in 55.2% of patients, with Grade ≥ 3 seen in 2.5% (n = 2). Other notable AEs include infections (including COVID-19) in 64% of patients. Among 134 patients with R/R B-cell NHL at all dose levels, the ORR and CR were 68.6% and 56.6% with a median DOR of 25.9 months [149]. At a median follow up of 24 months, the median PFS was 9.9 months, and the median OS was 20.4 months. Better responses were seen in 2L, no prior CAR-T, and transformed FL subgroups, unsurprisingly. This is a particularly encouraging combination as it attempts to overcome T-cell exhaustion that has been observed as mechanism of resistance to single agent bispecific therapy. Englumafusp enhances T-cell activation, while limiting expansion of exhausted T-cells. Pharmacodynamics have demonstrated an expansion of activated and effector memory CD8 + T cells leading to deep molecular responses based on circulating tumor-derived DNA (ctDNA) evaluation (by CAPP-Seq) [149, 154].

### Lenalidomide-based combinations

The most promising lenalidomide-based combination includes the ViPOR regimen which includes venetoclax, ibrutinib, prednisone and obintuzumab (NCT03223610). This combination inhibits multiple known lymphoma pathways, acknowledging that lymphoma progression cannot be easily treated by targeting only one pathway. A phase 1b-2 study enrolled 60 patients with R/R B-cell lymphomas (n = 50 with DLBCL) [150]. A high percentage had DHL/THL (40%) and post CAR-T relapse (40%). The ORR and CR rate were 54% and 38%, respectively, with a median time to response of 0.6 months [150]. Response rates were similar for patients with DHL/THL with an ORR/CR rate of 67%/53% for patients with *MYC*/*BCL2* rearrangements. The 2 year PFS and OS rates for the overall DLBCL population were 34% and 36%. Given the combination of 6 different anti-lymphoma agents, there was a high incidence of TEAE. Hematologic adverse events of any grade occurred in more than 90% of patients with grade 3 or 4 neutropenia, thrombocytopenia, and anemia observed in 52%, 45%, and 25% of patients, respectively. Serious adverse events occurred in 42% of the patients. Dose reductions and delays owing to toxic effects occurred in 17% and 25% of the patients, respectively, and five patients prematurely discontinued the study intervention owing to an unacceptable adverse event or intercurrent illness. While this regimen is likely too toxic for the majority of patients, the durable response rates in certain high-risk populations such as DHL/THL and patients relapsed post CAR-T are promising and may be beneficial for a subset of patients.

Zanubrutinib has also been investigated in combination with lenalidomide in a phase 1 study limited to Chinese patients (*n* = 66) (NCT04436107). The majority of patients had stage III-IV disease (83%). With a median follow up of 16.5 months, the ORR was 58% and CR rate was 42% for patients treated at RP2D [148]. The median PFS was 5.5 months, and the median OS has not yet been reached. The median DOR was 14.9 months. No dose limiting toxicities occurred. A grade > 3 TEAE was seen in 74.2% of all patients across different dose levels, with the most common AEs being neutropenia (57%), thrombocytopenia (15.2%), anemia (16.7%) and hypokalemia (10.6%). Adverse events leading to discontinuation, dose interruption or dose reduction occurred in 10.6%, 65.2%, and 10.6% of patients, respectively, which was similar in patients treated at RP2D.

Tafasitamab and lenalidomide are also being investigated in combination with zanubrutinib or tazemetostat in the SWOG S2207 clinical trial (NCT05890352).

Loncastuximab is also being investigated in combination with venetoclax for R/R LBCL after 2 prior lines of therapy (NCT05053659), based on preclinical data suggesting synergism with venetoclax [147]. Among 14 response evaluable patients treated so far, the ORR and CR rates were 57% and 43%. Adverse events were consistent with single agent safety data with no new safety signals seen. Dose expansion is ongoing.

## Management of patients with relapsed/refractory DLBCL

For the over one third of DLBCL patients who will relapse following frontline therapy, there are now more options than ever. The approval of therapies with immunotherapeutic and molecular mechanisms of action are huge improvements over cytotoxic chemotherapy. With all the recently approved therapies a subset of patients able to achieve CR may experience a durable remission for > 2 years, an outcome not observed with historical cytotoxic chemotherapy in the relapsed setting. However, none of these therapies work for all patients, and a high proportion of patients experience progression within the first 6 months of starting these new therapies. This highlights the importance of predictive biomarkers of both response and resistance to guide treatment selection and sequencing of these novel therapies.

All patients suspected of relapse, especially following frontline treatment, should undergo biopsy to confirm disease. It is not uncommon for other conditions (e.g., infections, sarcoidosis) to mimic lymphoma relapse. Additionally, patients may relapse with an indolent lymphoma, even if not seen on initial pathology. It is important to assess the expression of CD19 and CD20 on the relapse biopsy specimen to aid in treatment selection, as many of the recently approved therapies target one of these two cell surface markers. There is emerging data that target antigen expression can impact efficacy for some of these therapies (epcoritamab, glofitamab), while others have demonstrated efficacy regardless of target antigen expression (loncastuximab-tesirine, brentuximab-vedotin). Improving upon our techniques for antigen expression and defining clinically relevant thresholds are needed in order to optimize sequencing and treatment selection.

DLBCL is a molecularly heterogeneous and complex disease making precision medicine approaches challenging currently. However, testing with next generation sequencing (NGS) using commercially available platforms (Tempus, FoundationOne) may be useful in certain cases. NGS testing may provide insight into biological pathways driving disease progression. Several groups have identified high-risk factors based on NGS including gene panels to personalize therapy in DLBCL [155–158]. However, unlike solid tumors, NGS testing is not currently able to provide biomarker-driven treatment strategies given the lack of single driver mutations in DLBCL. Currently, NCCN guidelines recommend considering NGS testing at diagnosis. The 2022 WHO and ICC classifications do not recommend NGS testing in DLBCL [159, 160].

**Fig. 3:**
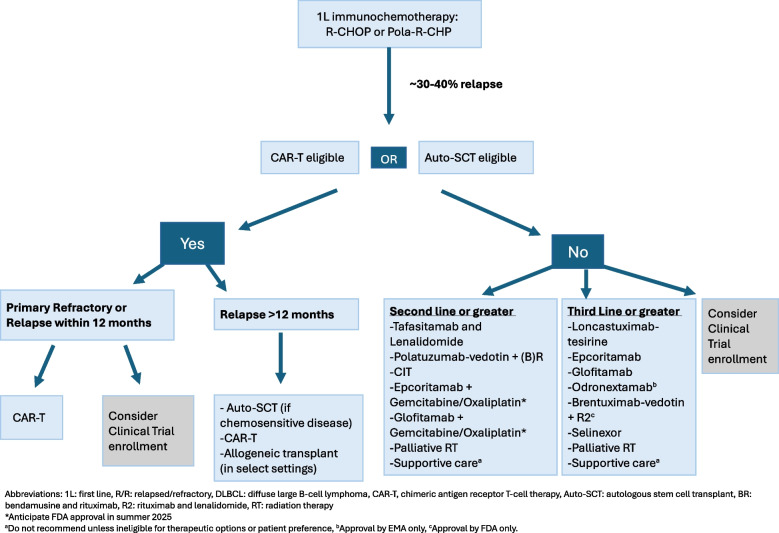
Treatment algorithm for R/R DLBCL**.** Current treatment paradigm for CAR-T/auto-SCT eligible and ineligible R/R DLBCL patients

**Fig. 4:**
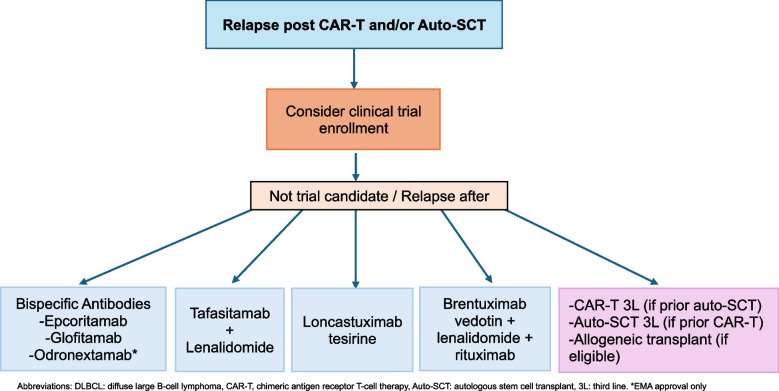
Treatment algorithm for DLBCL after CAR-T or auto-SCT relapse

For all patients, but especially those with suspected primary refractory disease, it is imperative to concurrently refer to a clinician with expertise in cellular therapy for determination of eligibility for either auto-SCT or CAR-T therapy. Logistic considerations for CAR-T and auto-SCT both take time and can be a barrier for those with aggressive disease or high-risk features. Choice of auto-SCT or CAR-T depends on timing of relapse post CAR-T and sensitivity to chemotherapy/bridging therapy (Fig. 3). For cellular therapy or transplant-ineligible patients or those relapsing after CAR-T/auto-SCT, treatment selection for relapsed disease must consider patient factors (comorbidities, location) and disease features (rapidity of disease progression, cell of origin, cell surface expression at relapse). Novel therapies are likely to be more efficacious and better tolerated for most patients (Fig. 4). However, limitations exist in side effect profile and time to response. For example, patients with comorbidities such as heart failure or kidney disease may have difficulty with loncastuximab which is associated with edema—including peripheral edema and pleural effusions. Time to response (**TTR**) can be another limitation as most of these therapies have first TTR of 1.4–2 months, limiting use in patients with rapidly progressive disease. Patients with refractory disease or early relapse remain a challenging subgroup of patients to treat as they often have rapidly progressive disease, but responses to chemotherapy will be transient. Radiation therapy is a useful treatment modality for many of these patients.

As all novel therapies have been approved in last 5 years, there is limited data on sequencing of available therapies. Additionally, clinical trials are now investigating combination therapies, which is likely to be efficacious and beneficial given the aggressive nature of DLBCL but may not be the right approach for older or unfit patients and will further complicate treatment selection and sequencing in the relapsed space.

## Conclusions

### How will we approach R/R DLBCL treatment in the future?

There continue to be many ongoing trials in the frontline space trying to improve upon R-CHOP or Pola-R-CHP. Notable trials include the incorporation of the bispecific antibodies epcoritamab and glofitamab to R-CHOP/Pola-R-CHP, and chemo-free or risk adapted strategies, such as CAR-T with axi-cel or rapcabtagene autoleucel (NCT03960840) for high-risk patients and the SMART STOP trial [161–165]. If any of these treatment paradigms, or other ongoing studies, show an improvement over the current standard of care, this will impact treatment selection in the relapsed setting. Given heterogeneous outcomes with CAR-T and bispecific antibodies in the relapsed setting, it is expected that a substantial proportion of patients will still experience relapse. Loss of CD19 and/or CD20 will impact drug development as many currently approved therapies are targeting one of these biomarkers. Measurements of residual disease utilizing ctDNA that can measure disease down to a threshold of 10^–6^ are being investigated in numerous trials and is emerging as a more sensitive method for response assessment compared to imaging [166]. CtDNA response during treatment has been associated with improved PFS. Measurable residual disease (MRD) positivity at end of treatment has been associated with inferior PFS and predictive of future relapse [167, 168]. This testing has the potential to identify patients prior to overt systemic relapse and prior to the development of symptoms, which has the greatest potential for benefit in patients with biologically aggressive and often more difficult to treat subsets of DLBCL (e.g., HGBCLs, plasmablastic lymphoma). We anticipate this will evolve to be incorporated into standard practice for response assessment and is another step forward for reducing invasive testing for DLBCL. In the future, ctDNA may either replace or complement imaging as the first step in workup for patients presenting with symptoms concerning for relapse. Challenges still remain in the cost, access to these tests for patients being treated in the community, and the absence of baseline ctDNA detection in some patients. Hopefully the future will also see the development of precision medicine approaches which has so far been limited, despite a deep knowledge of biological pathways driving lymphoma progression [169–173]. Combination treatment strategies will likely be needed given the interplay of multiple biological pathways driving DLBCL disease progression. With the large number of clinical trials ongoing for both frontline and relapsed DLBCL, it continues to be an exciting time for lymphoma with many changes anticipated to the standard of care in the next decade.

## Data Availability

No datasets were generated or analysed during the current study.

## Abbreviations

DLBCL: Diffuse large B-cell lymphoma
CAR-T: Chimeric antigen receptor T-cell therapy
Auto-SCT: Autologous stem cell transplant
NHL: Non-Hodgkin lymphoma
Pola-R-CHP: Polatuzumab-vedotin in combination with rituximab, cyclophosphamide, doxorubicin, and prednisone
IPI: International Prognostic Index
PFS: Progression free survival
OS: Overall survival
R-CHOP: Rituximab, cyclophosphamide, doxorubicin, vincristine and prednisone
HDT: High dose chemotherapy
CAR: Chimeric antigen receptor
Axi-cel: Axicabtagene ciloleucel
ORR: Overall response rate
CR: Complete response
3L: Third line
Liso-cel: Lisocabtagene maraleucel
R/R: Relapsed/refractory
BsAbs: Bispecific antibodies
IgG: Immunoglobulin G
BiTE: Bispecific t-cell engager
CNS: Central nervous system
CRS: Cytokine release syndrome
ICANS: Immune effector cell-associated neurological syndrome
DoCR: Duration of complete response
AE: Adverse events
G: Grade
IV: Intravenous
C: Cycle
D: Day
PJP: Pneumocystis jirovecii pneumonia
IVIG: Intravenous immunoglobulin
DHL/THL: Double-hit and triplet lymphoma, DLBCL with *MYC* and *BCL2* rearrangements
ANC: Absolute neutrophil count
ADC: Antibody–Drug Conjugates
Pola: Polatuzumab vedotin
MMAE: Monomethyl auristatin E
Pola-(B)R: Pola with bendamustine and/or rituximab
ABC: Activated b-cell
GCB: Germinal center b-cell
Lonca: Loncastuximab-tesirine
GGT: Gamma-glutamyl transferase
DOR: Duration of response
TEAEs: Treatment emergent adverse events
Tafa: Tafasitamab
Len: Lenalidomide
NK: Natural killer
HCT-CI: Hematopoietic cell transplantation-specific comorbidity index
NRM: Non relapse mortality
PR: Partial response
2L: Second line
Tisa-cel: Tisagenlecleucel
PMBCL: Primary mediastinal B-cell lymphoma
FL: Follicular lymphoma
MZL: Marginal zone lymphoma
ADLs: Activities of daily living
IADLs: Independent ADLs
CIRS-G: Cumulative Illness Rating Scale-Geriatric
HIV: Human immunodeficiency virus
RT: Radiation therapy
SOC: Standard of care
EFS: Event free survival
HR: Hazard ratio
4L: Fourth line
Allo: Allogeneic
DLT: Dose limiting toxicities
RP2D: Recommended Phase 2 dose
BV: Brentuximab vedotin
IHC: Immunohistochemistry
CELMoD: Cereblon E3 ligases
MR: Magrolimab and rituximab
GemOx: Gemcitabine and oxaliplatin
EZH2: Enhancer of zeste homolog 2
R-ICE: Rituximab, ifosphamide, carboplatin and etoposide
IRC: Independent review committee
R2: Lenalidomide and rituximab
BTKi: Bruton tyrosine kinase inhibitors
ctDNA: Circulating tumor-derived DNA
NGS: Next generation sequencing
TTR: Time to response
MRD: Measurable residual disease
